# WHO Global Antimicrobial Resistance Surveillance System (GLASS) for monitoring bloodstream infections due to *Candida*: findings from a demonstration study

**DOI:** 10.1016/j.eclinm.2026.104019

**Published:** 2026-06-23

**Authors:** Daniel Marcano Zamora, Sergey Eremin, Pilar Ramon-Pardo, Agustina Forastiero, Marcelo Galas, Daniel A. da Matta, Diego Nobrega, Zlatina Dobreva, Methee Chayakulkeeree, Jong Hee Shin, Susana Zurita, Arunaloke Chakrabarti, Anuradha Chowdhary, Ana Alastruey-Izquierdo, Susana Cordoba, Jana Welna, Nelesh P. Govender, Alexandre Alanio, Arnaldo Lopes Colombo, Ivana Mareković, Ingibjorg Hilmarsdottir, Silvia Bertagnolio, Carmem L. Pessoa-Silva

**Affiliations:** a Antimicrobial Resistance Department, World Health Organization, Geneva, Switzerland; b AMR Special Program. Communicable Diseases Prevention, Control and Elimination Department, Pan American Health Organization/WHO, Washington, D.C., USA; c Rodolphe Mérieux Laboratory, Rio Branco, Brazil; d Faculty of Veterinary Medicine, University of Calgary, Calgary, Canada; e Nuffield Department of Primary Health Sciences, University of Oxford, Oxford, United Kingdom; f Department of Medicine, Mahidol University, Bangkok, Thailand; g Department of Laboratory Medicine, Green Cross Laboratories, Gwangju, Republic of Korea; h National Reference Laboratory for Mycology, National Center for Public Health, National Institute of Health, Lima, Peru; i Doodhadhari Burfani Hospital and Research Institute, Haridwar, India; j Department of Medical Microbiology, Vallabhbhai Patel Chest Institute, University of Delhi, India; k Mycology Reference Laboratory, Instituto de Salud Carlos III, Madrid, Spain; l Instituto Nacional de Enfermedades Infecciosas Dr. Carlos G Malbrán, Buenos Aires, Argentina; m Antoni Jurasz University Hospital No. 1, Bydgoszcz, Poland; n Wits Mycology Division, Faculty of Health Sciences, University of the Witwatersrand, Johannesburg, South Africa; o Hospital Saint-Louis, Paris, France; p Antimicrobial Resistance Institute of São Paulo, Universidade Federal de São Paulo, São Paulo, Brazil; q University Hospital Centre Zagreb, Zagreb, Croatia; r Landspitali - National University Hospital of Iceland, Reykjavík, Iceland; s Federal University of Rio de Janeiro, Rio de Janeiro, Brazil

**Keywords:** GLASS, Candidemia, AMR

## Abstract

**Background:**

Antimicrobial resistance (AMR) in *Candida* species is an emerging global health threat. This study aims to document the implementation of standardized surveillance tools from the Global Antimicrobial Resistance and Use Surveillance System (GLASS) and to describe the distribution and antifungal resistance profiles of *Candida* species in bloodstream infections generated through the study outputs.

**Methods:**

The GLASS-Fungi pilot was a multicentre observational demonstration study where twenty-four laboratories were invited to collect and share *Candida* bloodstream infections (BSIs) data with the World Health Organization (WHO) using a standardized surveillance protocol and WHONET software. Data were collected for patients with laboratory-confirmed *Candida* bloodstream infections between January 2017 and July 2021. Participating sites were trained to collect, deduplicate, and report clinical and microbiological data. Patient demographic and microbiological data were summarized using descriptive statistics. The percent distribution of *Candida* species and resistance profiles were analysed with a 95% confidence interval (CI) calculated using robust standard errors clustered at the site level. Participating sites shared experiences with using the standardised GLASS surveillance tools and procedures through structured feedback forms and consultative meetings. This study was not designed to support causal inference or population-level generalization; analyses are descriptive and intended to illustrate outputs generated through pilot surveillance implementation.

**Findings:**

Fourteen laboratories from 13 countries contributed data from 3447 patients with candidemia. Overall, *Candida albicans* was the predominant species (37.6%; 95% CI: 33.8–41.5). *C. albicans* was most common in the Americas, Europe, and Africa, whereas *Candida tropicalis* was more prevalent in Southeast Asia. *Candida auris* was detected by two laboratories in Southeast Asia and Africa. Among isolates with interpretable antifungal susceptibility testing (AFST) results and corresponding established breakpoints, 13.6% (95% CI: 10.1–17.2%) were resistant to at least one antifungal, with fluconazole resistance highest among *C. parapsilosis* isolates (29.7%; 95% CI: 18.2–41.2). Challenges to scaling up fungal AMR surveillance globally included limited fungal laboratory testing capacity, restricted access to antifungal susceptibility testing, and lack of sustained funding, particularly in low and middle-income countries.

**Interpretation:**

The study was a global, multi-centre initiative to systematically collect and report surveillance data on *Candida* BSIs using standardised data collection tools and reporting procedures. The study identified major training capacity and infrastructure gaps. Addressing those is an essential step for anticipating and responding to emerging invasive fungal infections.

**Funding:**

This work was supported by the United States Centers for Disease Control and Prevention.

**Research in context**

**Evidence before this study**

In 2015, the World Health Assembly recognized antimicrobial resistance (AMR) as a global health threat, and member-states pledged to collaborate in a global action plan to address antimicrobial resistance. A major component of this global action plan is conducting surveillance to better estimate and monitor this public health problem. In 2015, the World Health Organization implemented the Global Antimicrobial Resistance and Use Surveillance System (GLASS) to collect surveillance data on priority bacterial pathogens; however, with the rapid emergence of drug resistance in *Candida auris* and other fungal pathogens, member states increasingly recognized the public health threat of antimicrobial-resistant fungal pathogens. The main body of evidence available prior to this study primarily comprised data from the SENTRY Antimicrobial Surveillance Program focused on high-income countries (HICs) and findings from various ad-hoc national and sub-national surveillance studies. These sources, while valuable, highlight a gap in systematic, region-specific data, particularly from low- and middle-income countries, underscoring the need for more comprehensive and globally representative surveillance efforts.

**Added value of this study**

This multicentre observational demonstration study documents the implementation of standardised GLASS surveillance tools and reporting procedures for *Candida* bloodstream infections (BSIs), and provides a descriptive summary of *Candida* species distribution and antifungal susceptibility testing (AFST) results generated through the pilot. Fourteen laboratories from 13 countries contributed data from 301 surveillance sites and 3447 patients with candidemia, with data collected for patients with laboratory-confirmed *Candida* BSIs between January 2017 and July 2021.

This study provides evidence supporting the feasibility of collecting and reporting fungal AMR surveillance data through GLASS in settings with fungal laboratory expertise, while also identifying operational challenges related to data extraction, conversion, upload, data-sharing agreements, laboratory capacity, and sustained funding. The findings showed variation in *Candida* species distribution across participating geographic regions. Among isolates with interpretable AFST results and established breakpoints, 13.6% were resistant to at least one antifungal, with fluconazole resistance highest among *Candida parapsilosis* isolates at 29.7%. These findings should be interpreted as outputs from a pilot surveillance implementation, rather than as population-level regional or global estimates.

**Implications of all the available evidence**

The growing threat of antimicrobial-resistant fungal infections justifies sustained global surveillance. The next step after the pilot is to scale up capacity to ensure reporting comes from a representative sample, not just those with existing capabilities.

## Introduction

In 2015, the World Health Assembly recognised antimicrobial resistance (AMR) as a global health threat, and Member States pledged to collaborate on a global action plan to address it.^1^ A major component of the plan is AMR surveillance to generate evidence on and monitor this public health problem. Early efforts focused on monitoring antimicrobial-resistance in eight bacterial pathogens through the Global Antimicrobial Resistance and Use Surveillance System (GLASS) launched in 2015.^2^ By the end of 2024, 138 countries, three territories and areas had joined the GLASS, with over 100 reporting AMR data for 2023.

The rapid global emergence of drug-resistant *Candidozyma auris* (synonym: *Candida auris*) and other fungal pathogens causing invasive infections in humans^3^, ^4^, ^5^ has intensified calls for systematic monitoring of antifungal resistance.^6^^,^^7^ In 2019, the United States's Antibiotic Resistance threat report raised concerns regarding some fungal pathogens.^8^ In 2022, the World Health Organization (WHO) published the first Fungal Priority Pathogen List^9^ that includes six *Candida* species and yeasts formerly classified as *Candida*, with *Candida auris* classified in the “critical priority group”. Although surveillance initiatives such as the SENTRY Antimicrobial Surveillance Program track trends in species distribution and resistance rates over time,^10^ they rely primarily on sentinel sites in well-resourced settings in the regions of the Americas, Europe, and Asia–Pacific, and provide limited clinical and epidemiological information. Consequently, substantial knowledge gaps persist regarding the burden and resistance patterns of invasive fungal infections in many world regions, with data from low- and middle-income countries particularly lacking.

GLASS has progressively expanded to include surveillance of AMR in invasive fungal pathogens causing bloodstream infections (BSIs), in response to the increasing incidence of invasive *Candida* infections, its emerging antifungal resistance, and associated treatment challenges. In 2019, WHO published the GLASS Early Implementation Protocol for Inclusion of *Candida* spp. [hereinafter referred to as “GLASS *Candida* BSI Surveillance Protocol”], which established standardised methods for monitoring BSIs caused by *Candida* species and yeasts formerly classified as *Candida*, aligned with established data collection methods for bacterial BSIs and integrated with existing GLASS activities. Following its publication, WHO developed supporting implementation tools, including standardized data variables for collection at the time of blood sampling, WHONET configurations adapted for fungal data capture and management (WHONET version 21.9.15), and a dedicated module within the GLASS Information Technology (IT) platform.

The aim of this study is twofold: to document the implementation of standardized GLASS surveillance tools and reporting procedures for *Candida* bloodstream infections across multiple international sites, and to provide a descriptive summary of *Candida* species distribution and antifungal susceptibility results generated through this study; the latter are intended as an exploratory outputs of the surveillance system rather than a population-level epidemiological estimate. The interpretation of species-level data should be considered in the context of recent taxonomic revisions based on molecular phylogenetic analyses, which have led to the reclassification of several clinically relevant yeasts formerly assigned to the genus *Candida* into other genera within *Saccharomycotina*.^11^, ^12^, ^13^ In this manuscript, the term *Candida* species refers to species within the genus *Candida* as well as related yeasts previously classified as *Candida*.

## Methods

### Technical collaborative platform to participate in GLASS-Fungi

Laboratories with known mycology expertise in all regions of the world were mapped in collaboration with all WHO Regional Offices and the WHO AMR Collaborating Centre Network members.^14^ The mapping exercise conducted in 2018 identified academic, public health or clinical expert laboratories with capacities for *Candida* species isolation, identification and antifungal susceptibility testing. These formed a Technical Collaborative Platform and were invited to assist WHO in developing and pilot-testing standards for monitoring AMR in BSIs due to *Candida* (Table 1). Nine laboratories contributed to both the development of the GLASS Candida BSI Surveillance Protocol and participation in GLASS-Fungi. An additional nine laboratories contributed only to protocol development, while six laboratories participated exclusively in the study by collecting, sharing, and analyzing AMR surveillance data on *Candida* BSIs.

**Table 1:** Roles of institutions contributing to the development of the GLASS *Candida* spp BSI Protocol and implementation of the GLASS-Fungi Pilot.

Institution name	Institution location (country)	Support the development of the GLASS *Candida* spp BSI protocol	Support implementation of the GLASS-fungi pilot	Provided feedback on the application of the surveillance tools	Provided surveillance data	Number of sites contributing surveillance data	Number of *Candida* spp. isolates from BSIs
Instituto Nacional de Enfermedades Infecciosas “Dr. Carlos G Malbrán”	Argentina	X	X	X	X	29	213
Universidade Federal de São Paulo	Brazil	X	X	X	X	1	105
University Hospital Centre Zagreb, Department of Clinical and Molecular Microbiology	Croatia	–	X	X	X	16	200
Hopital Saint-Louis	France	–	X	X	X	1	33
Dept. of Microbiology, Landspitali - The National University Hospital	Iceland	–	X	X	X	1	47
Postgraduate Institute of Medical Education and Research, Chandigarh	India	X	X	X	X	1	210
Vallabhbhai Patel Chest Institute, University of Delhi	India	–	X	X	X	1	96
Instituto Nacional de Salud	Peru	X	X	X	X	6	59
Antoni Jurasz University Hospital No 1	Poland	–	X	X	X	2	114
Chonnam National University Hospital	Republic of Korea	X	X	X	X	1	80
National Institute for Communicable Diseases	South Africa	X	X	X	X	92	1175
National Centre for Microbiology, Instituto de Salud Carlos III	Spain	X	X	X	X	1	66
Siriaj Hospital, Mahidol University	Thailand	X	X	X	X	1	98
U.S. Centers for Disease Control and Prevention, Mycotic Diseases Branch	United States of America	X	X	X	X	148	1011
National Institute of Health	Pakistan	–	X	X	–	–	NA
Aga Khan University	Pakistan	X	–	–	–	–	NA
King Saud bin Abdulaziz University for Health Sciences	Saudi Arabia	X	–	–	–	–	NA
Iranian Hospital -Dubai	United Arab Emirates	X	–	–	–	–	NA
University Hospital of Cologne	Germany	X	–	–	–	–	NA
Helsinki University Hospital	Finland	X	–	–	–	–	NA
Canisius Wilhelmina Hospital	Netherlands	X	–	–	–	–	NA
Public Health England National Mycology Reference Laboratory	United Kingdom	X	–	–	–	–	NA
Innsbruck Medical University	Austria	X	–	–	–	–	NA
European Center for Disease Prevention and Control	Sweden	X	–	–	–	–	NA

Abbreviations: GLASS, Global Antimicrobial Resistance and Use Surveillance System; BSI, Bloodstream infection; X, participated; -, not participated; NA, Not applicable.

### Patient identification, microbiological testing and data reporting procedures

The GLASS-Fungi pilot was a multicentre observational demonstration study that collected clinical and microbiological data from patients with suspected BSI, as determined by the treating physician, and with laboratory-confirmed isolation of any *Candida* species from blood specimens^15^ collected between January 2017 and July 2021. The detailed data collection and reporting methods are described in the GLASS *Candida* BSI Surveillance Protocol and summarized as follows.^15^ At each participating surveillance site, a blood sample was collected from patients with a suspected candidemia and tested at the surveillance site's laboratory. Surveillance sites captured patient information including the patient's age, sex, infection origin, and the hospital department where the patient was treated using a standardised form distributed to all pilot sites. Infection was classified as healthcare-associated or community-acquired in line with GLASS definitions.^15^ Infections were considered to be “healthcare-associated” if patients had been hospitalised for more than two calendar days at the time of specimen collection, or “community-acquired” for patients seeking care at an outpatient clinic or in the case of patients hospitalised for two calendar days or less at the time of specimen collection.

Fungal identification was performed according to commonly used methods including Analytical Profile Index (API) 20 or 32C, BD Phoenix, Vitek®2, matrix-assisted laser desorption/ionization-time of flight mass spectrometry (MALDI-TOF), molecular methods, and sequencing of rDNA genes depending on the laboratory. Antifungal susceptibility testing (AFST) methods included broth microdilution using the reference antifungal susceptibility method guidelines from the Clinical & Laboratory Standards Institute (CLSI) and European Committee on Antimicrobial Susceptibility Testing (EUCAST), Sensititre YeastOne™, Vitek®2, and ATBFungus,. Laboratories reported the identified *Candida* species and the AFST results, including minimal inhibitory concentration (MIC) values, and categorical interpretation of susceptibility.

The following data collection tools and reporting procedures in line with the GLASS *Candida* BSI Surveillance Protocol were used to ensure the quality and integrity of the data.^15^ WHONET and BacLink configurations capturing all variables in the patient data form were developed and implemented across all participating institutions (Supplementary Material 1). Participating institutions had to enter the data into WHONET,^16^ either manually or through conversion through BacLink, preparing a standardised file compatible with the WHO GLASS IT platform. A specific module in the GLASS IT platform was developed to collect 32 variables, including fourteen mandatory variables (COUNTRY, YEAR, HCF_ID, PATIENT_ID, AGE, GENDER, PATIENTTYPE, LABORATORYCODE, SAMPLE_DATE, ISOLATEID, SPECIMEN, PATHOGEN, ANTIBIOTIC, and PATIENTCOUNTER). The platform would reject the file and display a blocking error if a mandatory variable was missing, empty, or if incorrect coding was used. All variables had to be included in the submitted file, even if the variable was not mandatory and the field was left blank. For non-numeric mandatory variables, an ‘Unknown’ category was included to account for missing information.

As part of the pilot, 17 training sessions were provided to the participating laboratories on data collection and management using the WHONET software as well as on the GLASS IT platform. Training materials consisting of slide sets and an operational guide outlining standardised procedures for preparing fungal data for submission to the GLASS platform were also provided.

Participating laboratories applied a deduplication strategy in accordance with the GLASS *Candida* BSI Surveillance Protocol standards. Deduplication was performed per patient, per species, per calendar year. For each patient, only the first positive blood isolate of a given *Candida* species within a calendar year was included in the analysis. Subsequent isolates of the same species from the same patient within the same year were excluded. If different Candida species were identified from a single patient's blood culture (mixed candidemia), then each species was included in the surveillance data; therefore, the same patient could contribute more than one isolate if different species were identified or if isolates were reported in different calendar years. Prior to transmitting data to WHO, all personal identifiers that could be used for patient identification were removed. Missing or incomplete data and validation checks were conducted to assess whether AFST result interpretations were inconsistent with CLSI or EUCAST reference guidelines.^17^, ^18^, ^19^ In such cases, WHO requested participating laboratories to review and resubmit any missing or inconsistent data.

### Analysis of the distribution and resistance profiles of *Candida* species

Patient demographic (age, sex) and clinical (hospital admission department) characteristics were analysed as a percentage in each category out of the total samples collected. The percentage of samples analysed according to different laboratory fungal identification, AFST and reference guidelines used was also calculated. Antifungal resistance analyses were restricted to species identified at the sensu stricto level for which established breakpoints were available. Three AFST categorical interpretations (1- Susceptible; 2- Intermediate or Susceptible, increased exposure or Susceptible dose-dependent; and 3- Resistant) were assigned for the *Candida* species with established breakpoints according to the institution's reference guidelines. Data were analyzed at the isolate level. Prior to analysis, datasets were screened for missing values; incomplete observations were excluded using pairwise deletion, and no imputation procedures were implemented. Because hospital sites contributed between 1 and 210 isolates, with several sites (n = 167) contributing a single isolate, the data structure was considered to be unbalanced and unsuitable for stable estimation in three-level hierarchical models; accordingly, proportions and corresponding 95% confidence intervals (CIs) were estimated using binomial regression models with cluster-robust standard errors specified at the site level to account for within-site correlation. All analyses were conducted in R. Data were further validated through removal of duplicate or inconsistent records, verification of AFST interpretations, and restriction of resistance analyses to isolates with complete and interpretable results and established breakpoints. These steps resulted in minor changes to the analytical dataset, which are reflected in the reported estimates.

### Assessment of the practical challenges encountered during the GLASS-Fungi pilot

Through consultative online meetings, participating laboratories provided unstructured feedback on the clarity and usefulness of the GLASS *Candida* BSI Surveillance Protocol for routine fungal surveillance as well as on the data reporting procedures employed during the pilot. In addition, a structured online form was used to systematically collect feedback on the experiences and challenges with i) using the standardised electronic data reporting form, ii) converting data via the BacLink module from WHONET, and iii) creating the export file (to the GLASS IT platform) using WHONET. Participating laboratories were also asked to comment on their experiences with navigating and submitting surveillance data to the GLASS IT platform (Supplementary Material 2). Quantitative responses to multiple-choice questions were summarised by calculating the frequency of responses within each category. Given the exploratory nature of the pilot evaluation, qualitative feedback from both consultative meetings and open-ended survey responses was summarised descriptively by identifying recurrent issues and grouping them into thematic categories through team discussion at WHO.

Feasibility was defined as the ability of participating laboratories to implement standardised GLASS data collection, management, and reporting procedures, assessed across three domains: data submission, data quality, and operational performance.

### Ethical aspects and data governance

Each participating institution was responsible for ensuring that data collection, use, storage, and submission to WHO complied with its institutional policies and applicable national, local, and/or public health ethics and data-governance requirements. Before submitting data, participating institutions signed a Letter of Participation and Data Sharing confirming that all necessary ethical approvals or authorizations for piloting the protocol had been obtained in accordance with institutional policies, that applicable data-protection requirements had been met, and that data were submitted to WHO in anonymized format. Approval or reference numbers were not requested in these letters and were not centrally collected by WHO; therefore, approval or reference numbers are not available for reporting by individual site. Individual informed consent was not obtained for the central analysis because the pilot used routinely collected surveillance and laboratory data, involved no direct patient contact, intervention, additional specimen collection, or change to patient management, and only anonymized data were submitted to WHO. Where required by local ethics, public health, or institutional governance procedures, the use and submission of anonymized surveillance data were authorized or the requirement for individual informed consent was waived. Before data transmission, participating institutions removed personal identifiers and used coded patient and isolate identifiers. Participating laboratories were trained on standardized data-management procedures, including patient de-identification, deduplication, data validation, and secure submission through the GLASS IT platform. The collection, analysis, publication, and dissemination of data presented in this manuscript are in line with WHO data principles.^20^

### Role of the funding source

This work was supported by the United States Centers for Disease Control and Prevention. The funder provided financial support for the GLASS-Fungi pilot. CDC-affiliated experts provided advisory technical input during the development of the GLASS *Candida* spp. BSI Protocol and manuscript development; no additional involvement of funding sources to declare. The corresponding author had final responsibility for the decision to submit for publication.

## Results

### Surveillance data characteristics

Data from 3447 patients across 301 surveillance sites from fourteen laboratories in 13 countries were included in the analysis; as participation in the GLASS-Fungi pilot was voluntary and based on convenience sampling, findings reflect data from participating surveillance sites and are not intended to be globally representative. The number of sites contributing surveillance data submitted by each ranged from 1 to 148 (Table 1). Most surveillance sites (275 out of 301, 91.4%) were overseen by four national laboratories in charge of national *Candida* BSI surveillance. Each laboratory reported data on a median number of 103 patients (ranging from 31 to 1118). The duration of the surveillance period ranged from 8.5 months to 4.2 years. The surveillance start and end dates across the fourteen laboratories ranged from November 2020 to July 2021 for the shortest period, and from January 2017 to March 2021 for the longest period.

Among participating laboratories, MALDI-TOF was the most frequently used method for fungal identification (applied to 64.2% of all isolates), followed by Vitek®2 (19.9%) (Table 2). CLSI Broth microdilution (applied to 71.9% of all AFST) and Vitek®2 (11.7%) were the most common methods for AFST. CLSI guidelines were used to interpret 98.4% of all AFST.

**Table 2:** Characteristics of patients and laboratory methods in the GLASS-Fungi Pilot, 2017–2021.

Characteristic	n (%)^a^
**Patient and surveillance characteristics**	
**Year of specimen collection**	
2017	19 (0.6%)
2018	1015 (29.4%)
2019	1643 (47.8%)
2020	674 (19.6%)
2021	96 (2.8%)
**WHO region**	
Americas	1385 (40.2%)
African	1173 (34.0%)
South East Asian	407 (11.8%)
European	403 (11.7%)
Western Pacific	79 (2.3%)
**Country-income group**	
Low and middle income	1963 (57.0%)
High income	1484 (43.0%)
**Age**	
<1 year	574 (17.9%)
1–4	184 (5.8%)
5–17	123 (3.8%)
18–49	766 (23.9%)
50–64	657 (20.5%)
65 or older	899 (28.1%)
**Sex**	
Male	1805 (55.4%)
Female	1451 (44.6%)
**Hospital department**	
Adult ICU	552 (30.5)
Emergency	303 (16.7%)
Neonatal or Pediatric unit^b^	184 (10.2%)
Surgery	79 (4.4%)
Other^c^	693 (38.3%)
**Laboratory methods**	
**Identification method (n = 3507 isolates)**	
MALDI-TOF	2253 (64.2%)
Vitek	697 (19.9%)
API 20 or 32 C	68 (1.9%)
BD Phoenix	5 (0.2%)
Sequencing	2 (0.1%)
Conventional	1 (<0.1%)
Other	106 (3.0%)
Method not reported	375
**Antifungal susceptibility testing method (n = 20,847 tests)**	
Broth microdilution CLSI	12,709 (71.9%)
Broth microdilution EUCAST	19 (0.1%)
Vitek	2065 (11.7%)
Gradient diffusion	1233 (7.0%)
Sensititre YeastOne™	810 (4.6%)
ATBFungus	8 (<0.1%)
Other	830 (4.7%)
Method not reported	3173
**Reference guidelines (n = 20,847 tests)**	
CLSI	20,521 (98.4%)
EUCAST	322 (1.5%)
Other	4 (<0.1%)

Abbreviations: GLASS, Global Antimicrobial Resistance and Use Surveillance System; n, count; CLSI, Clinical and Laboratory Standards Institute; EUCAST, European Committee on Antimicrobial Susceptibility Testing; ICU, intensive care unit; MALDI-TOF, matrix-assisted laser desorption/ionization time-of-flight; API, Analytical Profile Index.

a Excludes missing data for age (n = 244), sex (n = 191), hospital department (n = 1636), identification method (n = 375), and antifungal susceptibility testing method (n = 3173).

b Includes Pediatric ICU, Neonatal ICU, Pediatrics and Neonatology.

c Includes Coronary Care, Medicine, Trauma, Oncology, Hematology and Obstetrics.

### Demographic and clinical characteristics

Most patients with candidemia were older adults (≥65 years: 28.1% out of 3447) or aged 50–64 years (20.5%) (Table 2). Infants under 1 year of age also represented a substantial proportion (17.9%). Overall, 55.4% of patients were male. Information on hospital department was available for 53% of candidemia patients, with the adult intensive care unit (ICU) being the most common location (30.5%) of admission among those reported.

### *Candida* spp. distribution and resistance profiles

**Fig. 1:**
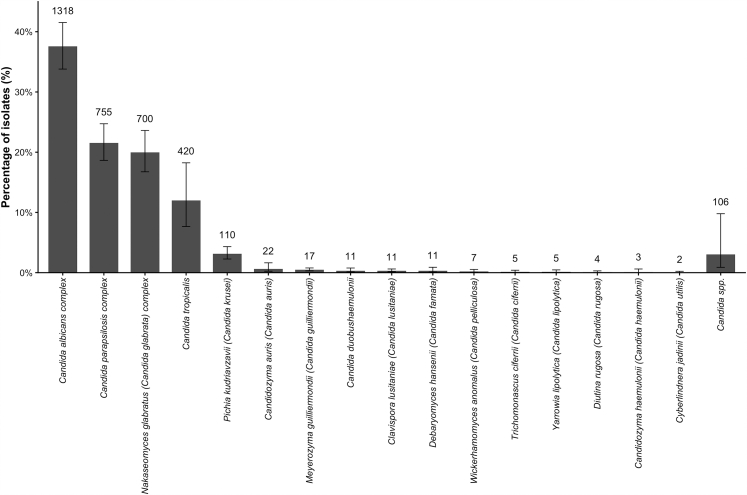
Distribution of *Candida* species from GLASS-FUNGI pilot data, 2017–2021. GLASS, Global Antimicrobial Resistance and Use Surveillance System.

The results presented below reflect the final validated analytical dataset following data cleaning and verification procedures described in the Methods. 3507 *Candida* isolates were reported from 3447 patients between 2017 and 2021. *Candida albicans* complex was the most common species globally (n = 1,318, 37.6%; 95% CI: 33.8–41.5), followed *by Candida parapsilosis* complex (n = 755, 21.5%; 95% CI: 18.6–24.7), *Nakaseomyces glabratus* (synonym: *Candida glabrata*) complex (n = 700, 20.0%; 95% CI: 16.7–23.6), *Candida tropicalis* (n = 420, 12.0%; 95% CI: 7.7–18.2) and *Pichia kudriavzevii* (synonym: *Candida krusei*) (n = 110, 3.1%; 95% CI: 2.3–4.3) (Fig. 1). *C. albicans* complex included 1273 isolates identified as *C. albicans sensu stricto*, 14 isolates identified as *Candida dubliniensis* and 31 isolates reported at complex level. *C. parapsilosis* complex included 736 isolates identified as *C. parapsilosis sensu stricto,* 10 isolates as *Candida metapsilosis,* and 9 isolates as *Candida orthopsilosis*. The five most common species complexes accounted for more than 94% of all candidemia cases reported, with other species individually accounting for less than 1%. *Candida auris* was reported by only two laboratories in the GLASS-Fungi pilot data (n = 22, 0.6%; 95% CI: 0.2–1.6).

**Fig. 2:**
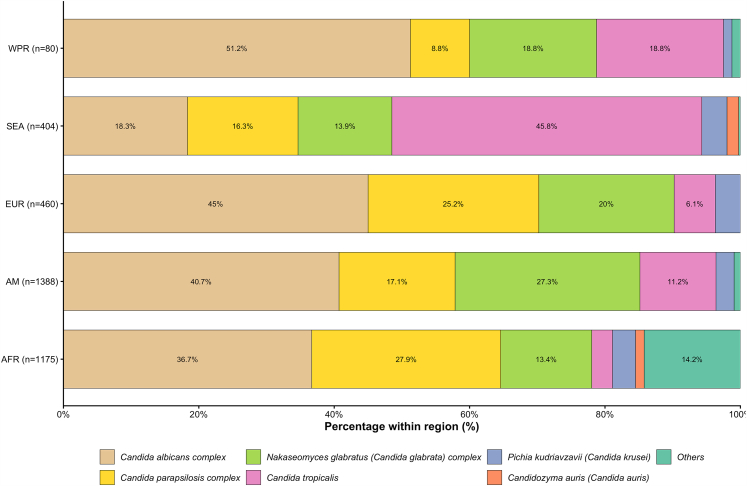
Distribution of *Candida* species in World Health Organization regions, GLASS-Fungi pilot, 2017–2021. GLASS, Global Antimicrobial Resistance and Use Surveillance System; AFR, African Region; AM, American region; EUR, European Region; SEA, South East Asia Region; WPR, Western Pacific Region. “Other” includes other *Candida* species as well as isolates that were not speciated and were reported as *Candida* spp. American region includes participating institutions from Argentina (29 contributing sites), Brazil (1 contributing site), Peru (6 contributing sites), and the United States of America (148 contributing sites). European region includes participating institutions from Croatia (16 contributing sites), France (1 contributing site), Iceland (1 contributing site), Poland (2 contributing sites), and Spain (1 contributing site). African region includes a participating institution from South Africa (92 contributing sites). Western Pacific region includes a participating institution from Republic of Korea (1 contributing site). Southeast Asia region includes participating institutions from India (2 contributing sites) and Thailand (1 contributing site). Data were generated from a convenience sample within a pilot implementation and do not constitute regionally representative surveillance data.

Species distributions differed by geographic region. Whereas *C. albicans* was the most common species in Africa (36.7%; 95% CI: 31.1–42.2), the Americas (40.7%; 95% CI: 37.2–42.2) and Europe (45.0%; 95% CI: 39.3–50.7), *C. tropicalis* was more prevalent in Southeast Asia accounting for 45.8% (95% CI: 36.9–54.7) (Fig. 2). *Candida auris* was detected by two laboratories in Southeast Asia and Africa.

**Fig. 3:**
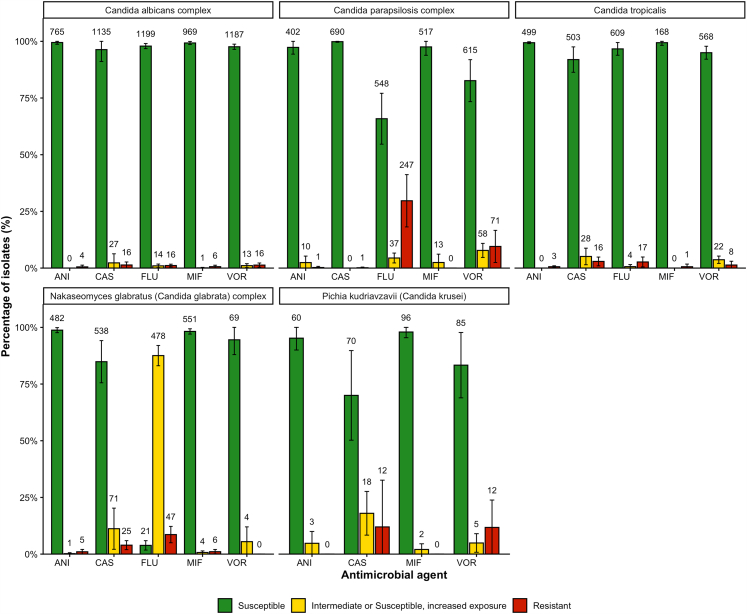
Antifungal susceptibility testing (AFST) results for *Candida* species with breakpoints, GLASS-Fungi pilot, 2017–2021. ANI: Anidulafungin; CAS: Caspofungin; FLU: Fluconazole; MIF: Micafungin; VOR: Voriconazole. Antifungal susceptibility testing results shown are restricted to species sensu stricto. Interpretive categories are based on clinical breakpoints defined by the reference guidelines applied by the reporting laboratory. Fluconazole results for *Candida krusei (Pichia kudriavzevii)* are not shown due to intrinsic resistance.

Categorical interpretation of resistance according to AFST breakpoints are presented in Table 3 and summarized in Fig. 3. Overall, among isolates with interpretable AFST results and corresponding established breakpoints, 13.6% (95% CI: 10.1–17.2%) were resistant to at least one of the following antifungal drugs: anidulafungin, caspofungin, fluconazole (excluding *C. krusei*), micafungin, or voriconazole. Among the major species–antifungal combinations, the highest resistance proportion was observed for fluconazole in *C. parapsilosis* (29.7%; 95% CI: 18.2–41.2).

**Table 3:** Antifungal susceptibility testing results by *Candida* species, GLASS-Fungi Pilot, 2017–2021.

Species	Drug	n tested	n susceptible	n intermediate or SDD	n resistant	% R
*Candida albicans*	Anidulafungin	769	765	0	4	0.5%
	Caspofungin	1178	1135	27	16	1.4%
	Fluconazole	1229	1199	14	16	1.3%
	Micafungin	976	969	1	6	0.6%
	Voriconazole	1216	1187	13	16	1.3%
*Candida parapsilosis*	Anidulafungin	413	402	10	1	0.2%
	Caspofungin	691	690	0	1	0.1%
	Fluconazole	832	548	37	247	29.7%
	Micafungin	530	517	13	0	0.0%
	Voriconazole	744	615	58	71	9.5%
*Candida tropicalis*	Anidulafungin	502	499	0	3	0.6%
	Caspofungin	547	503	28	16	2.9%
	Fluconazole	630	609	4	17	2.7%
	Micafungin	169	168	0	1	0.6%
	Voriconazole	598	568	22	8	1.3%
*Nakaseomyces glabratus* (synonym: *Candida glabrata*)	Anidulafungin	488	482	1	5	1.0%
	Caspofungin	634	538	71	25	3.9%
	Fluconazole	546	478	47	21	3.8%
	Micafungin	561	551	4	6	1.1%
	Voriconazole	73	69	0	4	5.5%
*Pichia kudriavzevii* (synonym: *Candida krusei*)	Anidulafungin	63	60	3	0	0.0%
	Caspofungin	100	70	18	12	12.0%
	Fluconazole	–	–	–	–	Intrinsic
	Micafungin	98	96	2	0	0.0%
	Voriconazole	102	85	5	12	11.8%

Abbreviations: ANI, Anidulafungin; CAS, Caspofungin; FLU, Fluconazole; MIF, Micafungin; VOR, Voriconazole; R, Resistant; SDD, Susceptible, dose-dependent; n, number of isolates tested; GLASS, Global Antimicrobial Resistance and Use Surveillance System; CLSI, Clinical and Laboratory Standards Institute; EUCAST, European Committee on Antimicrobial Susceptibility Testing.

Notes: Results are restricted to isolates identified at species sensu stricto level with established CLSI or EUCAST breakpoints. Intermediate category includes isolates classified as Intermediate or Susceptible, increased exposure (EUCAST), or Susceptible, dose-dependent (CLSI). Fluconazole results for *Pichia kudriavzevii* (*Candida krusei*) are not shown due to intrinsic resistance.

### Feasibility and lessons learned from implementing the GLASS-Fungi pilot

#### Feasibility was assessed across three domains: data submission, data quality, and operational performance

##### Data submission feasibility

Fifteen laboratories provided structured feedback on data reporting procedures, of which all but one successfully submitted surveillance data to the GLASS platform. Most laboratories reported technical challenges related to data extraction from laboratory information management systems, conversion of national data into GLASS-compatible formats, and the data upload process. Overall, 78% of laboratories were able to convert their data using the BacLink module from WHONET. Two laboratories reported difficulties in obtaining data sharing agreements, which prevented data submission in one case. Additional issues included blocking errors due to missing mandatory variables, problems with access credentials, and incomplete data uploads.

##### Data quality feasibility

Overall, levels of missing data were low for key variables, including age (7.1%), sex (5.5%), pathogen identification method (10.7%), and AFST method (15.2%). Reasons for missing data included limited access to patient clinical records, incomplete reporting by laboratory personnel, and recording of categorical susceptibility interpretations without corresponding minimal inhibitory concentration values in laboratory information systems.

##### Operational feasibility

Eleven laboratories provided feedback on the implementation of the GLASS *Candida* BSI Surveillance Protocol. Eight reported that the protocol was clear and well defined, and all but one indicated that it was useful for strengthening fungal AMR surveillance. Reported challenges included perceived inconsistencies between the protocol and software requirements, and the need for more detailed guidance on data variables and reporting procedures. Satisfaction with training, technical support, and communication with WHO was generally high, with 73% of respondents rating these aspects as good or very good. Lower satisfaction was reported for the functionality of WHONET, the GLASS IT platform, and the data upload process. Laboratories also identified key requirements for successful implementation, including sustained funding, additional training on fungal diagnostics and data reporting, improved coordination between national AMR focal points and laboratories, and strengthened data management systems.

### Lessons learned

Taken together, these findings highlight several practical lessons for implementation. Most technical challenges related to data extraction, conversion, and submission were resolved during the pilot, indicating that iterative support and communication are critical for successful implementation. However, recurring issues—including inconsistencies between protocol specifications and software requirements, and difficulties with data conversion and sharing agreements—identify key operational bottlenecks. Participating laboratories emphasized the need for clearer guidance on data variables, additional training on data management and fungal diagnostics, and improvements in data conversion and upload processes. Enablers of successful implementation included strong institutional coordination, leadership commitment to surveillance and data sharing, and the availability of standardized data systems. Laboratories also highlighted the importance of sustainable funding and increased awareness of invasive fungal infections as prerequisites for scaling up surveillance.

## Discussion

The GLASS-Fungi pilot was a global, multi-centre initiative to systematically collect and report surveillance data on invasive fungal bloodstream infections and document lessons learnt from implementing standardised data collection methods and reporting procedures to inform global scale-up under the GLASS. The most prevalent fungal pathogen identified across 301 surveillance sites based on data from 13 countries was *C. albicans* complex (37.6%; 95% CI: 33.8–41.5). Resistance to at least one antifungal drug was identified in 13.6% (95% CI: 10.1–17.2%) of isolates with interpretable antifungal susceptibility testing results and established breakpoints, with fluconazole resistance being most notably detected in 29.7% (95% CI: 18.2–41.2) of *C. parapsilosis* isolates.

This study should be interpreted primarily as a pilot implementation of a standardized surveillance framework rather than as a formal epidemiological assessment. While the data allow for descriptive characterization of species distribution and antifungal susceptibility patterns within participating sites, they are not intended to provide fully representative estimates at regional or global levels. The data collected through this pilot showed notable geographic variations in the distribution of *Candida* species and reflected a similar AMR candidemia profile to that previously reported in single-center studies.^21^^,^^22^ *C. albicans* was the most common species in the Americas, Europe, and Africa, and *C. tropicalis* was most prevalent in Southeast Asia. *Candida auris* was reported by only two laboratories in Southeast Asia and Africa, although the prevalence and geographic spread of the species has increased globally.^23^, ^24^, ^25^ *C. auris* is classified at the highest threat level in both the WHO's Fungal Priority Pathogen List and the United States' 2019 antimicrobial threats report due to its multidrug resistance, impact on morbidity and mortality, and ability to easily spread in healthcare facilities.^8^^,^^9^ The global spread and rising prevalence of *Candida auris* since the implementation of this pilot further emphasizes the need for routine surveillance to monitor trends and spread of fungal AMR and guide public health action.^26^, ^27^, ^28^

The emergence of resistance in *C. parapsilosis* represents another serious concern. This pathogen has historically been reported as commonly susceptible to fluconazole. From 1997 to 2016, the SENTRY surveillance program reported global estimates of fluconazole resistance at 3.6%.^10^ Occasional investigations of fluconazole-resistant *C. parapsilosis* outbreaks were reported prior to 2019, highlighting the need to monitor its resistance. Since 2019, and potentially partly due to the COVID-19 pandemic, publications reporting on fluconazole-resistant *C. parapsilosis* have increased dramatically.^29^, ^30^, ^31^, ^32^, ^33^, ^34^, ^35^ The 29.7% frequency of fluconazole resistance detected in the GLASS-Fungi Pilot data is in line with the growing concern reported through single-site studies and highlights the need for coordinated global surveillance to monitor changes and trends in resistance alongside other *Candida* spp.

Although these findings are broadly consistent with published patterns of species distribution and antifungal resistance, they are not globally or nationally representative. Participation in the pilot was voluntary, and some countries contributed data on a small number of patients relative to their population size; in addition, certain regions were represented by only a few countries; as a result, the regional distribution of Candida species and associated resistance patterns cannot be considered fully representative. The observed distributions reflect the patient populations served by participating surveillance sites and may have been influenced by opportunistic sampling rather than systematic case ascertainment. Differences in data collection periods across laboratories further limit the ability to assess or adjust for temporal trends. Additional factors affecting interpretability include heterogeneity in fungal identification methods and antifungal susceptibility testing (AFST) practices across sites, which may affect comparability of results; also, the restriction of resistance analyses to isolates with interpretable susceptibility results and established breakpoints may influence the estimated proportion of resistance and limits comparability with studies using broader inclusion criteria. Finally, as the primary objective of the study was to assess operational feasibility, formal quantitative evaluation of inter-site reliability and measurement validity was not undertaken. These limitations should be considered when interpreting the descriptive resistance estimates.

Nevertheless, as a proof-of-concept study, the GLASS-Fungi pilot provides evidence supporting the feasibility of implementing standardised data collection and reporting procedures across participating sites, particularly with respect to data submission and data quality; however, feasibility varied across settings, with reported challenges related to data conversion, system usability, and resource constraints indicating that implementation remains context-dependent. WHONET was identified as a powerful data reporting component of the GLASS, compatible with national laboratory information management systems. Participating institutions reported several issues with the data conversion and upload processes, so that regular data quality checks and communication with submitting laboratories were crucial for ensuring that data were accurately submitted. Participating laboratories were generally satisfied with the trainings and technical support provided by WHO, regular meetings and communications, and the overall use of the protocol. Together with the limited amount of missing data for key variables reported during the pilot, this feedback provided supporting evidence for launching the GLASS Fungi component under the 2024 GLASS data call, accompanied by updated training materials and revised data preparation and submission procedures. As part of this for the 2023–2024 period, 16 countries submitted national surveillance on *Candida* species to GLASS underscoring the feasibility of scaling up the standardised protocol and reporting procedures.

Importantly, the success of this pilot relied on the technical expertise and capacities in advanced fungal diagnostics of the participating laboratories. MALDI-TOF was the most common tool for identification, and broth microdilution was the most common method used for AFST. However, access to these advanced methods remains limited in many laboratories worldwide.^36^, ^37^, ^38^, ^39^, ^40^ The pilot also highlighted gaps in AFST harmonisation and quality assurance, laboratory information system integration, and data management and analysis, needed for expanding fungal AMR surveillance globally. To overcome these challenges, pilot laboratories highlighted the need for funding, training, political commitment, and technical support from institutions with established expertise particularly to strengthen capacity and guide the implementation of surveillance microbiological, epidemiological and analytical methods in low- and middle-income countries. Importantly, the lessons learnt from this pilot will further inform the establishment of methodologies for broader fungal monitoring beyond the *Candida* species in the pilot.

The global scale-up of routine candidemia surveillance may be further challenged by limitations in some conventional and automated methods to reliably identify *Candida* at the species level, particularly for less common species. Conventional laboratory methods and some automated methods (including Vitek®2, BD Phoenix, and others) may fail to distinguish between *Candida* and related species in certain cases, necessitating the reporting of the isolate at the species complex level when identification is unclear. Even advanced methods such as MALDI-TOF can fail to identify rare fungal species. Current susceptibility breakpoints depend on species identification to determine isolate resistance to antifungals, and breakpoints are established for only the most common fungal species.

Surveillance is the backbone that guides and assesses the effectiveness of strategies to control and mitigate the impact of AMR on human health and development. In September 2024, during the 79th United Nations General Assembly, all countries unanimously agreed to a comprehensive chart of actions listed in the Political Declaration of the High-level Meeting on Antimicrobial Resistance.^41^ Among several commitments, the Declaration pledges to “strengthen national capacities for sustainable, sector-specific, integrated and interoperable surveillance systems for antimicrobial resistance and antimicrobial use, standards of diagnostics, laboratory information systems and networks, and other infrastructure to support the collection of nationally representative data on prevalence, antimicrobial resistance patterns…”. With an initial focus on candidemia, WHO GLASS aims to establish the first global system for aggregating national surveillance data on antimicrobial resistant invasive fungal infections. Despite the challenges, expanding national fungal AMR surveillance capacities and global monitoring are essential to anticipate the emergence of global public health threats.

While the UNGA Declaration signals political commitment, adequate funding and technical support will be needed to expand global AMR surveillance to fungal species. Furthermore, as shown in this pilot, the collaboration of technical institutions can be an effective lever, and more than ever, similar collaborative efforts will be needed to leverage global AMR invasive fungal surveillance. The target set by the UNGA Declaration that “at least 80 per cent of countries can test resistance in all bacterial and fungal GLASS pathogens by 2030” must be pursued.

## Contributors

DMZ, CLP, SE and SB conceived and coordinated the study. SE, CLP coordinated the development of the surveillance protocol. AAI, ArC, MC, ALC, SC, NPG, JHS, SZ, MG, PRP, AnC, AF participated in the development of the surveillance protocol. MC, JHS, SZ, ArC, AnC, AAI, SC, JW, NPG, AA, ALC, IM and IH collected and reported data. DDM, DN and DMZ performed data analysis and accessed and verified the underlying data. DMZ and ZD wrote the draft of the paper. All authors reviewed, commented and approved the final manuscript.

## Data sharing statement

Codebook and aggregate data by institution are available at (https://worldhealthorg-my.sharepoint.com/:f:/g/personal/dmarcano_who_int/EpYBK3bbOxJBhfrjfa9tVqABpySBjAHK-pzvS3kvx5S-8Q?e=pP3sYQ) immediately following publication with no end date to anyone who wishes to access the data.

## Declaration of interests

ALC reports honoraria for lectures and educational events from Mundipharma, Sandoz, Gilead, Knight, and IMMY; expert opinion fees from Mundipharma; travel support from Knight; and serves as President of the International Society for Human and Animal Mycology (ISHAM). AC reports honoraria from Pfizer, Glenmark, and Gilead. ZD reports previous employment at the World Health Organization and is currently a self-funded DPhil student at the University of Oxford who has received unrelated university support for travel and research expenses in the area of antimicrobial resistance. NG reports grants to his institution from UK NIHR, US CDC, UKRI-MRC, Gates Foundation, GAMRIF Failsafe, and Wellcome; unpaid participation on the ACACIA trial DSMB and the 5FC Crypto Project Advisory Board; unpaid leadership roles with FIDSSA and the End AIDS Action Group; and receipt of diagnostic kits for research work from IMMY delivered to his institution. PRP reports serving as a member of the Advisory Board for the International Master on International Health at the University of Oxford. AAI reports a grant from Scynexis to test the preclinical activity of a new antifungal compound, paid to her institution; honoraria from Gilead and Pfizer for educational talks; travel support from Gilead to attend ESCMID 2023, including flights and accommodation; participation on an advisory board for Basilea for a new antifungal compound in 2025; unpaid service as a member of the JPIAMR Scientific Advisory Board from 2022 to 2024; and unpaid leadership roles as President of the Spanish Society for Mycology and President of Women for Mycology. All other authors declare no competing interests.
