# Supplementary material for: WHO Global Antimicrobial Resistance Surveillance System (GLASS) for monitoring bloodstream infections due to Candida: findings from a demonstration study
Source: eClinicalMedicine. 2026 Jun 23;97:104019. doi: 10.1016/j.eclinm.2026.104019 (PMC13316650; doi:10.1016/j.eclinm.2026.104019)
Supplement: Supplementary Material [file mmc1.docx]

**Supplementary Material 1: BacLink and WHONET configuration**

To ensure standardized data processing across participating laboratories, a structured configuration was implemented using BACLINK for data conversion; the same parameters were used for the creation of a WHONET laboratory configuration that allowed data entry, data management and export into a comma-separated text file compatible with the GLASS IT fungal module.

The BACLink configuration mapped each laboratory’s raw export structure to the standardized microbiology data model of WHONET using the predefined variables described below. These same variables were incorporated into the resulting WHONET laboratory configuration and its data entry module to ensure consistency between imported and manually entered data.

Species-specific clinical breakpoints were predefined for *Candida* spp. and related yeasts to enable automated antifungal susceptibility interpretation. Standardized dictionaries were implemented for institutions, departments, organisms, antifungal agents, and infection sites to promote harmonized data entry and analytical comparability across sites. The configuration further included predefined institutional and departmental categories (e.g., intensive care units, pediatric services, surgical wards, transplant units, and outpatient clinics) to support consistent clinical stratification. The antimicrobial dictionary comprised antifungal agents aligned with both CLSI and EUCAST methodologies.

Supplementary Table 1: List of reported variables

| **Variable** | **Type** |
| --- | --- |
| COUNTRY | Coded value |
| YEAR | Numeric |
| HCF_ID | Text |
| HCF_TYPE | Coded value |
| HOSPITALUNITTYPE | Coded value |
| PATIENT_ID | String |
| AGE | Numeric |
| GENDER | Coded value |
| PATIENTTYPE | Coded value |
| DATEOFHOSPITALISATION_VISIT | Date |
| PATIENTCOUNTER | Numeric |
| LABORATORYCODE | Coded value |
| SAMPLE_DATE | Date |
| ISOLATEID | Text |
| SPECIMEN | Coded value |
| PATHOGEN | Coded value |
| ANTIBIOTIC | Coded value |
| SIR | Coded value |
| REFERENCEGUIDELINESSIR | Coded value |
| DISKLOAD | Text |
| RESULTETESTSIGN | Coded value |
| RESULTETESTVALUE | Numeric |
| RESULTETESTSIR | Coded value |
| RESULTZONEVALUE | Numeric |
| RESULTZONESIGN | Coded value |
| RESULTZONESIR | Coded value |
| RESULTMICSIGN | Coded value |
| RESULTMICVALUE | Numeric |
| RESULTMICSIR | Coded value |

**Supplementary Material 2: Structured Pilot Feedback Forms**

*GLASS Candida Pilot survey 1*

- Name: (TEXT)
- Country: (TEXT)
- Institution: (TEXT)
- Have you been able to upload your data for the pilot? (Yes/No)
  - If you answer is no, please select what is the possible problem
    - Issues with data sharing agreement (Yes/No)
    - Issues with the data extraction (Yes/No)
    - Issues with data conversion (Yes/No)
    - Issues with data upload (Yes/No)
    - Other issues (TEXT)
- Were you able to extract your data from your LIS system (Yes/No/Doesn´t apply)
  - If your answer is no, please explain your limitations and if you have any plans on how to circumvent this situation (TEXT)
- Have you installed the latest WHONET software version? (Yes/No)
- Were you able to convert your data using the BACLINK module from WHONET (Yes/No)
  - If you answer is no, please select what is the possible problem
    - Problems configuring BACLINK (Yes/No)
    - Problems with the conversion of variables, such as variables missing (Yes/No)
    - Other problems (TEXT)
- Were you able to configure WHONET according to your laboratory? (Yes/No)
- Were you able to create your GLASS export file using WHONET (Yes/No)
  - If you answer is no, please select what is the possible problem
    - Cannot generate a GLASS export file (Yes/No)
    - Cannot get the correct variables in the export file (Yes/No)
    - Cannot get all isolates into the export file (Yes/No)
    - Other problems (TEXT)
- Would you need additional WHONET trainings for configuration and data conversion? (Yes/No)
- Were you able to upload your data to the GLASS preproduction platform?
  - If you answer is no, please select what was the possible problem
    - Issues with your credentials (Yes/No)
    - The platform gives blocking errors that prevent for advancing on the upload process (Yes/No)
    - The platform gives an error that excludes some isolates from your files (Yes/No)
    - Other problems (TEXT)
- Additional comments: (TEXT)

*GLASS Candida Pilot survey 2*

- Name: (TEXT)
- Country: (TEXT)
- Institution: (TEXT)
- Do you consider that the document *GLASS early implementation protocol for the inclusion of* Candida *spp* presented in a clear manner? (yes/no)
- Did the *GLASS early implementation protocol for the inclusion of* Candida *spp* document provide a clear guidance to support the pilot phase of the AMR surveillance in *Candida* spp in countries? (yes/no)
  - If your answer is “No”, what do you think is missing in the document in terms of providing a clear guidance for countries? (TEXT)
- Do you consider the *GLASS early implementation protocol for the inclusion of* Candida *spp* document present clearly requirements and definitions for implementing AMR surveillance in *Candida* spp? (yes/no)
  - If your answer is “No”, what is missing in the document in terms of requirements and/or definitions for implementing AMR surveillance in *Candida* spp? (TEXT)
- Do you find the *GLASS early implementation protocol for the inclusion of* Candida *spp* useful for assisting with enhancing AMR surveillance system in your country? (yes/no)
  - If your answer is ‘No’, what do you think is missing in the document for you to find it useful for assisting with enhancing AMR surveillance system in your country? (TEXT)
- What do you consider would be necessary to increase the capacity and infrastructure in your country in terms of fungal identification and performing AFST? (TEXT)
- Based on your experiences during the pilot, what do you think would be required to implement the *GLASS early implementation protocol for the inclusion of* Candida *spp* in the national AMR surveillance system? (TEXT)
- Were you able to collect all the variables described in the *GLASS early implementation protocol for the inclusion of* Candida *spp* document during the pilot phase? (yes/no)
  - If your answer is “No”, please briefly explain the reasons (TEXT)
- Were there any important limitations or challenges regarding specimen collection, identification or AFST during the *GLASS early implementation protocol for the inclusion of* Candida *spp* pilot in your institution? (yes/no)
  - If your answer is “No”, please briefly explain (TEXT)
- Were you able to convert your data passing through software WHONET (Yes/No)
  - If you answer is no, please select what is the possible problem
    - Problems configuring BACLINK (Yes/No)
    - Problems with the conversion of variables, such as variables missing (Yes/No)
    - Other problems (TEXT)
- Were you able to create your GLASS export file using WHONET (Yes/No)
  - If you answer is no, please select what is the possible problem
    - Cannot generate a GLASS export file (Yes/No)
    - Cannot get the correct variables in the export file (Yes/No)
    - Cannot get all isolates into the export file (Yes/No)
    - Other problems (TEXT)
- Did you require WHONET trainings or any conference call with the WHO team for this pilot study? (Yes/No)
- Were you able to upload your data to the GLASS platform?
  - If you answer is no, please select what was the possible problem
    - Issues with the data extraction from your LIMS (Yes/No)
    - Issues with data conversion (Yes/No)
    - Issues with your credentials (Yes/No)
    - The platform gives blocking errors that prevent for advancing on the upload process (Yes/No)
    - The platform gives an error that excludes some isolates from your files (Yes/No)
    - Other problems (TEXT)
- From one (1) “poor”to five (5) “very good”, describe your experience about the following aspects of this pilot:
  - ( ) Data sharing mechanisms and agreements
  - ( ) Trainings and technical support from WHO team
  - ( ) WHONET software use
  - ( ) GLASS IT Platform
  - ( ) Update meetings and communications
  - ( ) Data upload process
  - ( ) Use of the *GLASS early implementation protocol for the inclusion of* Candida *spp* document
- Can you briefly share your experiences during this pilot testing? (TEXT)
